# Systemic regulation of nodule structure and assimilated carbon distribution by nitrate in soybean

**DOI:** 10.3389/fpls.2023.1101074

**Published:** 2023-02-06

**Authors:** Sha Li, Chengbin Wu, Hao Liu, Xiaochen Lyu, Fengsheng Xiao, Shuhong Zhao, Chunmei Ma, Chao Yan, Zhilei Liu, Hongyu Li, Xuelai Wang, Zhenping Gong

**Affiliations:** ^1^ College of Resources and Environment, Northeast Agricultural University, Harbin, China; ^2^ College of Agriculture, Northeast Agricultural University, Harbin, China; ^3^ College of Engineering, Northeast Agricultural University, Harbin, China

**Keywords:** nitrate, dual-root soybean, nodule structure, symbiosome, carbon distribution

## Abstract

**Background:**

The nitrate regulates soybean nodulation and nitrogen fixation systemically, mainly in inhibiting nodule growth and reducing nodule nitrogenase activity, but the reason for its inhibition is still inconclusive.

**Methods:**

The systemic effect of nitrate on nodule structure, function, and carbon distribution in soybean (*Glycine max* (L.) Merr.) was studied in a dual-root growth system, with both sides inoculated with rhizobia and only one side subjected to nitrate treatment for four days. The non-nodulating side was genetically devoid of the ability to form nodules. Nutrient solutions with nitrogen concentrations of 0, 100, and 200 mg L^-1^ were applied as KNO_3_ to the non-nodulating side, while the nodulating side received a nitrogen-free nutrient solution. Carbon partitioning in roots and nodules was monitored using ^13^C-labelled CO_2_. Other nodule responses were measured *via* the estimation of the nitrogenase activity and the microscopic observation of nodule ultrastructure.

**Results:**

Elevated concentrations of nitrate applied on the non-nodulating side caused a decrease in the number of bacteroids, fusion of symbiosomes, enlargement of the peribacteroid spaces, and onset of degradation of poly-β-hydroxybutyrate granules, which is a form of carbon storage in bacteroids. These microscopic observations were associated with a strong decrease in the nitrogenase activity of nodules. Furthermore, our data demonstrate that the assimilated carbon is more likely to be allocated to the non-nodulating roots, as follows from the competition for carbon between the symbiotic and non-symbiotic sides of the dual-root system.

**Conclusion:**

We propose that there is no carbon competition between roots and nodules when they are indirectly supplied with nitrate, and that the reduction of carbon fluxes to nodules and roots on the nodulating side is the mechanism by which the plant systemically suppresses nodulation under nitrogen-replete conditions.

## 1 Introduction

The effect of nitrate on N_2_ fixation in legumes is complex, and the underlying mechanism is not clear. Several studies have shown that the inhibitory effect of nitrate on nodule initiation and development in legumes depends on an interaction between nitrate and the autoregulation signal (Okamoto et al., 2009; Reid et al., 2011; Lin et al., 2018; Nishida et al., 2018). It is also thought that nitrate can accumulate in nodules, accelerating the senescence of the nodules (Cabeza et al., 2014). The subsequent metabolism of nitrite then produces nitric oxide, which easily combines with leghemoglobin to form nitrosyl leghemoglobin (LbNO), reducing the oxygen binding site of leghemoglobin and blocking the O_2_ supply (Becana et al., 1985; Minchin et al., 1989; Kanayama et al., 1990; Matamoros et al., 1999). Furthermore, the assimilation and reduction of nitrate in the plant require a sufficient energy source; thus, the proportion of carbohydrates available to nodules is reduced (Wasfi and Prioul, 1986; Fujikake et al., 2002).

Some studies have shown that supplying nitrates leads to carbon (C) competition between roots and nodules in legumes. Kouchi and Nakaji (1985) fed ^13^C to soybean (*Glycine max* (L.) Merr.) for 2.5 h and found that ^13^C was preferentially allocated to nodules compared to roots. In another study, a supply of 5 mM nitrate to soybean resulted in a 50% decrease in ^14^C radioactivity in nodules and a doubling of ^14^C radioactivity in roots (Fujikake et al., 2003b). Khan and Khan (1981) observed a similar phenomenon in pea (*Pisum sativum* L.). (Voisin et al. 2003a; Voisin et al. 2003b) concluded that nitrate inhibited nodule growth and reduced nodule dry weight, while ^13^C allocation was proportional to dry weight; thus, the proportion of ^13^C allocated to nodules was reduced.

Adequate C supply plays an important role in maintaining the stability of nodule structure. Supplying soybean roots with 5 mM nitrate under hydroponic conditions inhibited the expansion of infected cells in nodules (Fujikake et al., 2003b). When 50 mM nitrate was supplied to chickpea (*Cicer arietinum* L.) roots under sand culture conditions, infected cell nuclei disappeared, the bacteroids became sparse, and the nodule nitrogenase activity decreased (Sheokand et al., 1998). In studies of barrel medic (*Medicago truncatula*), trifolium (*Trifolium subterraneum* L.), lupin (*Lupinus albus* L. cv. Multolupa), and bean (*Phaseolus vulgaris*), nitrate supply reduced the number of infected cells and increased the number of uninfected cells in nodules; moreover, the bacteroids were irregularly shaped and decreased in number, the symbiosome membrane (SM) was destroyed, and the peribacteroid space (PBS) became larger (Dart and Mercer, 1965; De Lorenzo et al., 1990; Matamoros et al., 1999; Wang et al., 2020).

In previous studies on the relationships among nodule structure, C distribution and nitrate, nitrate was added directly to the roots, which failed to prevent the toxic effects caused by nitrate coming into direct contact with nodules. At the same time, experiments with split-rooted plants cannot avoid the effects of incomplete root systems caused by removal of the main root. We used grafting to prepare dual-root soybean plants with one side nodulating and another side non-nodulating to ensure the integrity of the root system on both sides. Meanwhile, we supply nitrate to the non-nodulating side, which excluded the toxic effects of nitrate on nodules. The hypothesis of this work is that roots and nodules compete for C during indirect nitrate exposure, which induces damage to nodule structure and down-regulation of N_2_ fixation. We measured the nodule nitrogenase activity and plant C distribution (^13^C labeling method), and observed the nodule structure. Our objective was to investigate the systemic effects of nitrate on nodule structure and assimilated C distribution by excluding the toxic effects of nitrate exposure and to provide new insights into the systemic inhibition of nodule N_2_ fixation by nitrate.

## 2 Materials and methods

This study was carried out in sand culture pots in 2019 at the Experimental Base of Northeast Agricultural University located in Xiangfang District, Harbin, Heilongjiang Province, China (geographical coordinates: 126°43′E, 45°44′N). Nodulating soybean (DongDa1 *G. max* L. cv.) and non-nodulating soybean (WDD01795, L8-4858 *G.*, a mutant of normal nodular soybean Clark, obtained from the Academy of Agricultural Sciences in China, Beijing) were used. The non-nodulating soybeans do not nodulate throughout the growth period, even if inoculated with rhizobia. Two varieties of soybeans were seeded into fine-sand medium and cultured in an illuminated growth chamber at 30°C for approximately 3 days. Nodulating and non-nodulating soybean seedlings were grafted together as previously described (Zhang et al., 2020). One week after grafting, the shoot of nodulating soybean was retained, and the shoot of non-nodulating soybean was cut off from the grafting site. Each pot contained two root systems sharing one shoot (see **Figure S1** for the detailed method for preparing dual-root soybean systems). At the VC stage (unfolded cotyledon stage), both root systems were inoculated with rhizobia as follows: field-grown soybean nodules that were frozen in the previous year were ground and added to the nutrient solution, with approximately 5 g of the ground nodule mass per liter. The inoculant was applied for five consecutive days. Before the VC stage, the plants were irrigated with distilled water once a day; from the VC stage to the V4 stage (fourth trifoliate leaf stage), the plants were irrigated with nutrient solution once a day, and from the V4 stage to the end of the experiment, the plants were irrigated with nutrient solution twice a day, in the morning and evening. The irrigation volume was 250 mL on each side of the root system. Nitrogen (N)-free nutrient solutions were prepared as previously described (Li et al., 2021), and the nutrient solution ingredients are listed in **Table S2**. The results of the previous study found that the effect of N concentration of 100 mg L^-1^ on nodulation was obvious (Lyu et al., 2019; Li et al., 2021). Therefore, in this experiment, KNO_3_ with N concentration of 100 mg L^-1^ and extreme N concentration (200 mg L^-1^) was selected to systemically analyze the effect of high nitrate concentrations on N_2_ fixation capacity of soybean nodules. While KNO_3_ was added to the non-nodulating side, K_2_SO_4_ was used to replace KNO_3_ on the nodulating side and in the control treatment to ensure an equal concentration of K^+^ in all the treatments. Stages were designated according to the description of Fehr et al. (1971).

### 2.1 Experimental treatments

The experiment included three treatments, designated N_0_, N_100_ and N_200_, between the VC and V4 stages. All the experimental materials were irrigated with a nutrient solution with N concentration of 14 mg L^–1^ KNO_3_ on both sides of the dual-root system. In the V4 stage, the N-free nutrient solution was added to both sides for N starvation over 10 days. After N starvation, in the R1 stage (42 days after grafting), the root systems subjected to the N_100_ and N_200_ treatments on the non-nodulating side were treated with a nutrient solution with a N concentration of 100 or 200 mg L^-1^ KNO_3_, and the nodulating sides of the dual-root systems were treated with the N-free nutrient solution. The N_0_ treatment was used as a control, and both sides were treated with the N-free nutrient solution. The experiment had a completely randomized design with three biological replications. After 4 days of treatment, the experimental materials of the three treatments were divided into two groups. One group was sampled for determination of nodule nitrogenase activity and light and electron microscopic observations, and the other group was subjected to a ^13^CO_2_ feeding.

^13^CO_2_ feeding was conducted following the method of Kouchi and Yoneyama (1984) with slight modifications. The assimilation chamber (1.2 m × 1.2 m × 1.0 m with acrylic sheets) was designed as a tightly closed system with a controllable CO_2_ generated device connected outside, and the air temperature and relative humidity were controlled at 27 ± 1°C and 60 ± 5%, respectively. The experiment was carried out under natural light on a sunny day. At the beginning of the feeding, ^13^CO_2_ was produced by adding 33.33 atom% Ba^13^CO_3_ and 1 mg L^-1^ HCl into the generated device. ^13^CO_2_ was imported into the assimilation chamber through the air inlet. The concentration of CO_2_ in the assimilation chamber was monitored continuously by an infrared C dioxide analyzer (Q-S151 Infrared CO_2_ Analyzer, Qubit Systems Inc., Canada), and the concentration of ^13^CO_2_ was maintained between 450 and 500 ppm (see **Figure S3** for the ^13^CO_2_ assimilation device). Feeding was performed for 8 h with sampling.

### 2.2 Sampling and determination

Nodule nitrogenase activity was measured as follows: the nodulating sides of the roots were washed with distilled water, and the nodule nitrogenase activity was measured by the acetylene reduction method described by Xia et al. (2017).

Light microscopy was performed as follows: six nodules of similar size within 6 cm of the grafting site were randomly chosen and cut out together with an adjacent portion of the root. Then, they were fixed, sliced (nodules on top and the connected roots on the bottom, cutting perpendicular to the roots from top to bottom), dehydrated, and embedded as described by Feder and O'Brien (1968), observed under a light microscope (Nikon Eclipse E100) and photographed using an imaging system (Nikon DS-U3).

Transmission electron microscopy (TEM) was performed as follows: six nodules of similar size within 6 cm of the grafting site were randomly chosen and cut out together with an adjacent portion of the root. According to the method described by Goodchild and Bergersen (1966), the nodules were sliced and fixed in 2.5% glutaraldehyde in 0.1 mol L^-1^ phosphate buffer (pH 7.0), washed with the same buffer, postfixed in 1% osmic acid buffer for 2 h, dehydrated by an acetone series, and embedded with Epon812 resin. The samples were polymerized at 60°C, and other operations were performed at room temperature. Sections were prepared with an ULTRACUT-E ultrathin slicer, stained in uranyl acetate-lead citrate and examined using a Hitachi H_7650 electron microscope.

Sampling and measurements of assimilated ^13^CO_2_ were conducted as follows: at the end of the ^13^CO_2_ feeding, soybean plants with dual-root systems were cut at the grafting site. The plants were divided into shoots and underground parts; the underground parts were divided into root samples from the non-nodulated side and root and nodule samples from the nodulated side. All the samples were washed with distilled water to remove sand and blot-dried with filter paper. The samples were then oven-dried at 65°C. After that, the samples were converted to ^13^CO_2_ in an elemental analyzer, and the ^13^C isotope abundance in each organ was determined by isotope mass spectrometry (Thermo-Fisher Delta V Advantage IRMS).

### 2.3 Data analysis

Distribution of C in samples was calculated according to the following formula:

^13^C Accumulation=W×C_T_× (^13^C_sample_ atom% - ^13^C_N_ atom%)

where W is the dry matter weight, C_T_ is the total C concentration, ^13^C_sample_ is the ^13^C abundance in the sample, and ^13^C_N_ is the natural ^13^C abundance.

IBM SPSS 21.0 (IBM Corp., Armonk, NY, USA), Origin 9.0 and Microsoft Excel were used to analyze the data and generate graphs. All data were tested for normality before one-way analysis of variance (ANOVA), and Duncan’s multiple range test was run for mean comparisons at a significance level of *p*<0.05.

## 3 Results

### 3.1 Effects of nitrate on nitrogenase activity in soybean nodules

**Figure 1:**
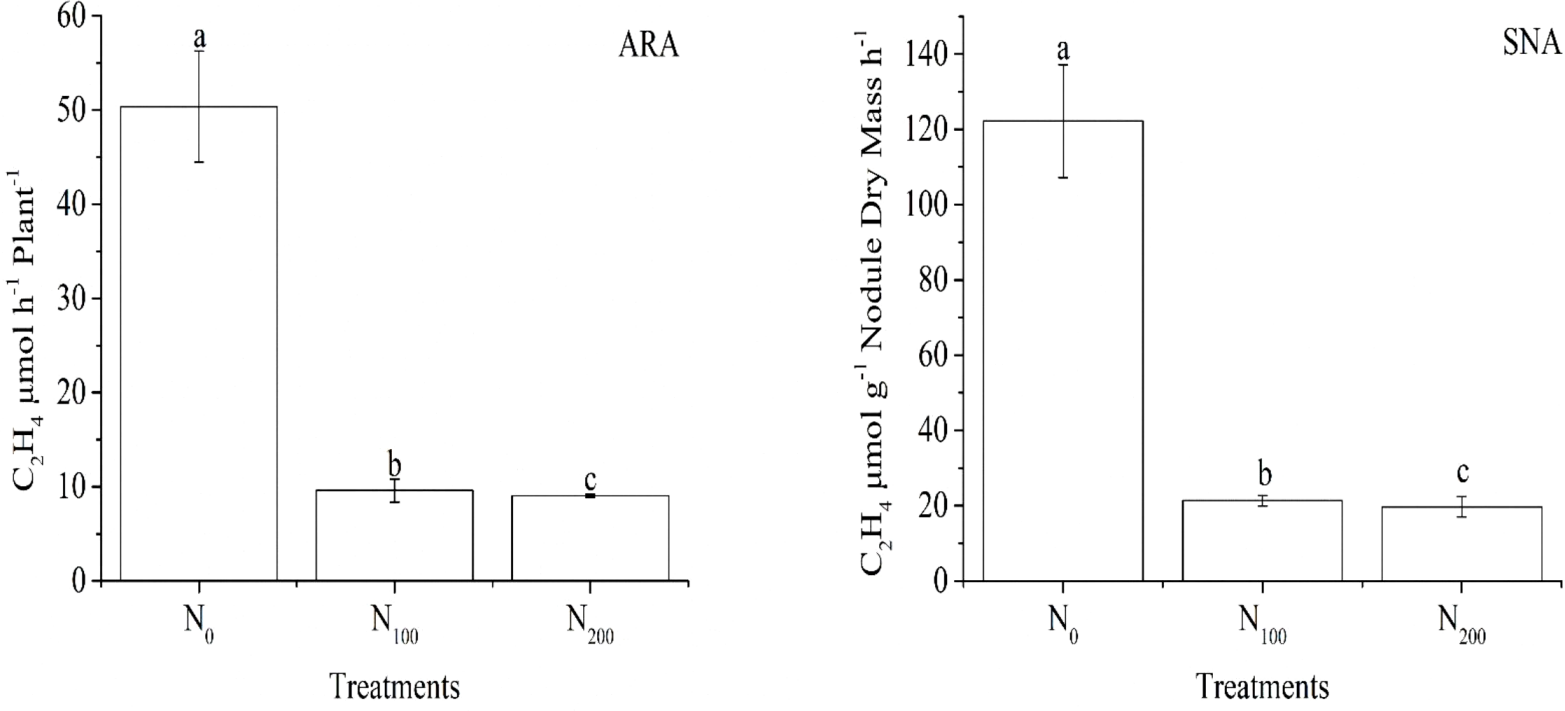
Acetylene reduction activity in μmol of ethylene formed per plant per hour (ARA) and specific nitrogenase activity per gram dry weight of nodules per hour (SNA) on the nodulating side of the dual-root soybean plants. The N_0_, N_100_, and N_200_ treatments represented the supply of KNO_3_ nutrient solution with N concentrations of 0, 100, and 200 mg·L^-1^ to the non-nodulating side, respectively, with N-free nutrient solution supplied to the nodulating side. Error bars are the standard error of three replicates. Different lowercase letters indicate a significant difference between the treatments at the 5% level.

The acetylene reduction activity in μmol of ethylene formed per plant per hour (ARA) and specific nitrogenase activity per gram dry weight of nodules per hour (SNA) were affected by the nitrate supply on the non-nodulating side in the same trend (**Figure 1**). The ARA and SNA in both the N_100_ and N_200_ treatments were more than 80% lower than those in the N_0_ treatment, and the ARA and SNA values between the N_100_ and N_200_ treatments were similar. This result indicated that the supply of nitrate on the non-nodulating side reduced nitrogenase activity on the nodulating side of dual-root soybean plants. In the experiment, when nitrate was supplied to the non-nodulating side, N-free nutrient solution was supplied to the nodulating side, the nodules were not exposed to nitrate, indicating that the nodule N_2_ fixation activity was systemically regulated by a certain mechanism in soybean plants.

### 3.2 Effects of nitrate on the anatomy of soybean nodules

#### 3.2.1 Microstructure of soybean nodules

**Figure 2:**
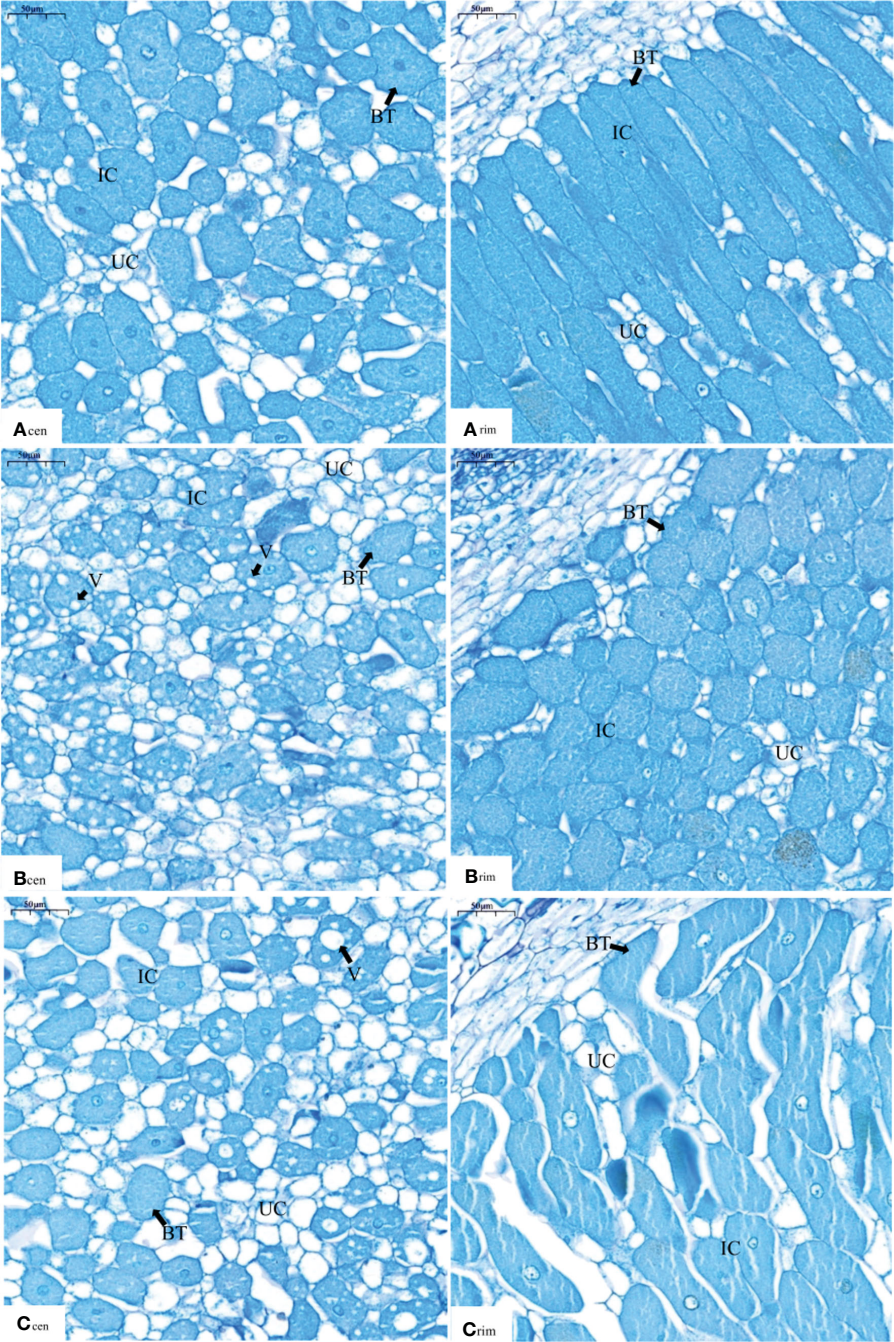
Light micrographs of nodules of dual-root soybean plants (×200). A_cen_, B_cen_ and C_cen_ are light micrographs of the central part of the nodules under the N_0_, N_100_, and N_200_ treatments, respectively. A_rim_, B_rim_, and C_rim_ are light micrographs in the edge part of nodules close to the roots under the N_0_, N_100_, and N_200_ treatments, respectively. IC represents infected cells, UC represents uninfected cells, BT represents bacteroids, and V represents vacuoles.

Supplying nitrate to the non-nodulating side resulted in changes in the nodule microstructure on the nodulating side (**Figure 2**). In the central part of the nodule, the infected cells were filled with bacteroids, and there were more infected cells than uninfected cells in the N_0_ treatment (**Figure 2**A_cen_). In the N_100_ treatment, a large number of vacuoles were produced in addition to bacteroids in the infected cells (**Figure 2**B_cen_). In the N_200_ treatments, the vacuoles were enlarged compared with those in the N_100_ treatment; the number of infected cells was significantly lower and the number of uninfected cells was higher than those in the N_0_ and N_100_ treatments (**Figure 2**C_cen_). The results indicated that, as the nitrate supply increased on the non-nodulating side, the number of infected cells in the central part of the nodules on the nodulating side decreased, the number of uninfected cells increased, and the vacuoles in the infected cells became enlarged.

In the edge part of the nodules close to the root, most of the infected cells were rod-shaped, the bacteroids in the infected cells were densely packed with bacteroids, and there was basically no gap between the infected cells (**Figure 2**A_rim_). In the N_100_ treatment, the infected cells were filled with bacteroids somewhat less densely and were mostly spherical in shape compared with those in the control N_0_ (**Figure 2**B_rim_). In the N_200_ treatment, most of the infected cells had irregular shape different from the shape of cells in the control N_0_ and the N_100_ treatment, while the number of uninfected cells appeared to be similar to the numbers observed in the control N_0_ and the N_100_ treatment (**Figure 2**C_rim_). At the same time, the vertical cracks through the infected cells and the large spaces between the cells apparent in **Figure 2**C_rim_ are likely to be due to mechanical damage to the nodule section, namely due to a stretching force that pulled the cells apart from each other in the horizontal direction. We think that this observation is artifactual because the contours resemble the drift of continents and clearly show which cell was mechanically separated from which other cells in the image. The results indicated that the structure of the infected cells close to the root changed as the concentration of nitrate supplied to the non-nodulating side increased.

#### 3.2.2 Ultrastructure of soybean nodules

**Figure 3:**
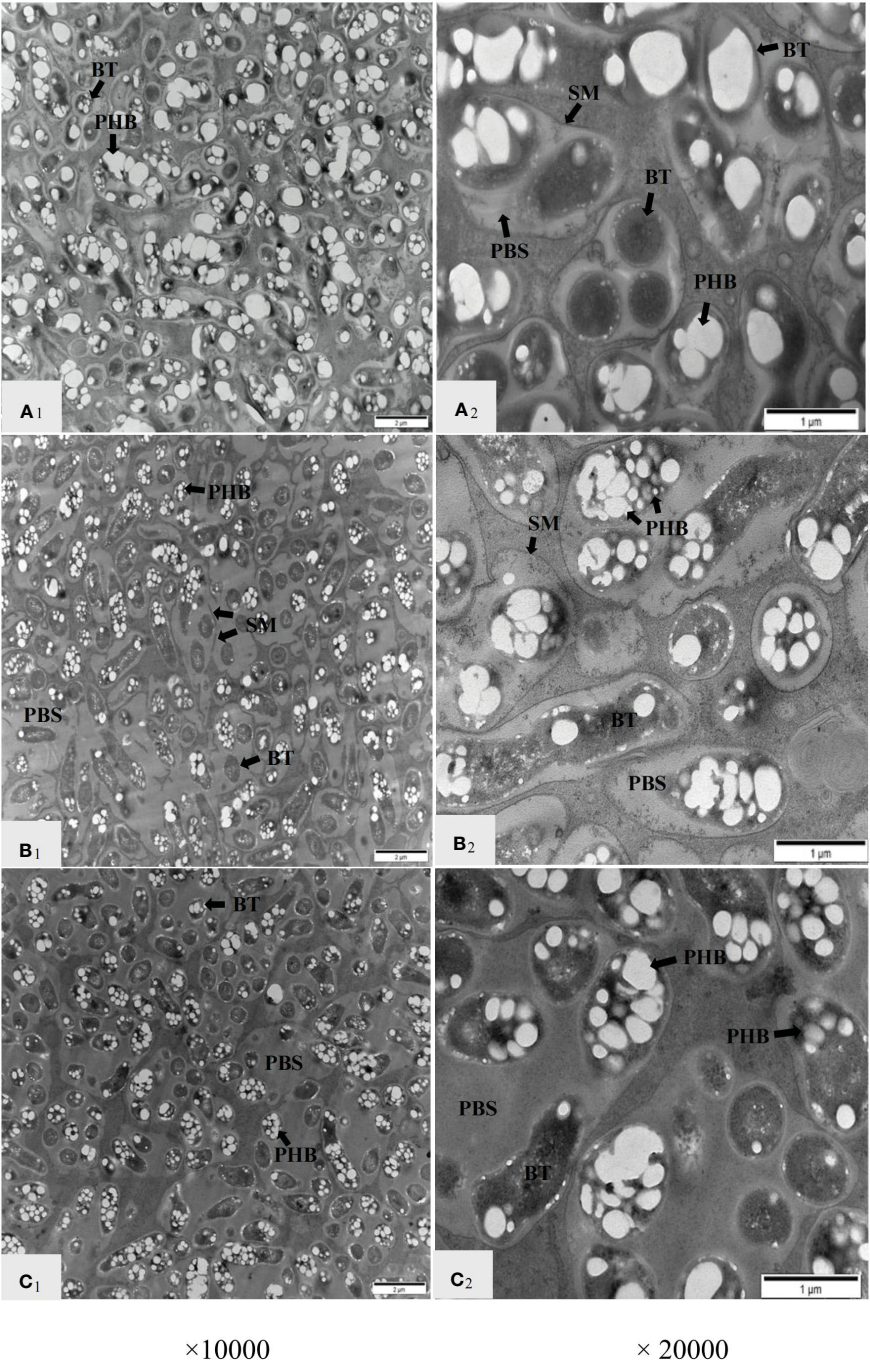
Transmission electron micrographs of infected cells in nodules on the nodulating side of dual-root soybean plants. A_1_ and A_2_ represent the N_0_ treatment; B_1_ and B_2_ represent the N_100_ treatment; C_1_ and C_2_ represent the N_200_ treatment. BT, bacteroids; SM, symbiosome membrane; PBS, peribacteroid space; PHB, polyhydroxybutyrate granules.

TEM was used to further observe the nodule structure, and it was found that the nitrate supply on the non-nodulating side had a great influence on the symbiotic structure of the infected cells on the nodulating side (**Figure 3**). In the infected cells in the N_0_ treatment, multiple bacteroids were wrapped with an SM to form a symbiosome (SB), and the SBs were positioned compactly (**Figure 3**A_1_). The SMs were clearly visible, the bacteroids in the SBs were dense, and the PBS between the bacteroids was very small. Most of the bacteroids were filled with large PHB granules, which appeared white and bright under TEM (**Figure 3**A_2_). Compared with the N_0_ treatment, some SBs were fused in the N_100_ treatment, and an incompletely degraded SM was observed in the fused SBs (**Figure 3**B_1_). The PBS became larger, the number of bacteroids decreased, the PHB granules in the bacteroids became smaller than those in the N_0_ treatment, and some of the PHB granules became translucent (**Figure 3**B_2_). In the N_200_ treatment, the number of PHB granules and the number of bacteroids were comparable with the N_100_ treatment. However, more PHB was present in the translucent form, and the PBS became larger. The SMs were less clearly outlined possibly indicating the onset of degradation, while the SBs were still well-distinguishable from the cytoplasm (**Figure 3**C_1_, C_2_). These results indicated that the supply of nitrate on the non-nodulating side led to a change in the symbiotic structure of the nodule-infected cells.

### 3.3 Effects of nitrate on assimilated C distribution in soybean plants

Dual-root soybean plants were fed with ^13^CO_2_ for 8 h after 4 days of nitrate supply on the non-nodulating side in the R1 stage, and we measured the dry weight of each organ (**Table 1**). The dry weights of the shoots in the N_100_ and N_200_ treatments were 9.6% and 17.8% higher than those in the N_0_ treatment, respectively (*P*<0.05). The dry weight of non-nodulating roots (Root_non_) in both the N_100_ and N_200_ treatments was 40.0% higher than that in the N_0_ treatment (*P*<0.05). The dry weight of roots on the nodulating side (Root_n_) and nodules was not significantly affected by changes in the concentration of nitrate supplied on the non-nodulating side. These results indicated that the short-term supply of high nitrate concentrations to the non-nodulating side promoted the growth of the roots on the non-nodulating side and of the shoots but had a weaker effect on the growth of roots and nodules on the nodulating side.

**Table 1:** Dry weights of dual-root soybean plants (g plant^-1^).

Organs	Shoot	Nodule	Root_n_	Root_non_
N_0_	7.3 ± 0.25 b	1.0 ± 0.03 a	1.2 ± 0.07 a	0.5 ± 0.03 b
N_100_	8.0 ± 0.34 ab	1.0 ± 0.09 a	1.2 ± 0.12 a	0.7 ± 0.00 a
N_200_	8.6 ± 0.23 a	1.0 ± 0.07 a	1.2 ± 0.06 a	0.7 ± 0.03 a

Root_n_ represented roots on the nodulating side, Root_non_ represented roots on the non-nodulating side. Values are means ± standard error (n=3). Different lowercase letters indicate a significant difference between the treatments in the same organ at the 5% level.

Nitrate supply on the non-nodulating side had little effect on the ^13^C abundance of the shoots but significantly increased the ^13^C abundance of non-nodulating lateral roots and significantly decreased the ^13^C abundance of nodulating lateral roots and nodules (**Table 2**). The ^13^C abundance of nodules in the N_100_ and N_200_ treatments was 8.3% and 16.7% lower than that in the N_0_ treatment, respectively (*P*<0.05). The ^13^C abundance of Root_n_ in the N_100_ and N_200_ treatments was 7.7% and 15.4% lower than that in the N_0_ treatment, respectively (*P*<0.05). The abundance of ${\text{Root}}_{\text{non}}^{13}$ C in the N_100_ and N_200_ treatments was 20.0% and 35.0% higher than that in the N_0_ treatment, respectively (*P*<0.05).

**Table 2:** ^13^C abundance of dual-root soybean plants (atom%).

Organs	Shoot	Nodule	Root_n_	Root_non_
N_0_	4.8 ± 0.02 a	2.4 ± 0.07 a	2.6 ± 0.15 a	2.0 ± 0.07 b
N_100_	4.8 ± 0.10 a	2.2 ± 0.10 ab	2.4 ± 0.07 ab	2.4 ± 0.15 ab
N_200_	4.8 ± 0.10 a	2.0 ± 0.06 b	2.2 ± 0.03 b	2.7 ± 0.15 a

Root_n_ represented roots on the nodulating side, Root_non_ represented roots on the non-nodulating side. Values are means ± standard error (n=3). Different lowercase letters indicate a significant difference between the treatments in the same organ at the 5% level.

Based on the dry weights (**Table 1**), ^13^C abundance (**Table 2**) and total C concentration (**Table S4**) of dual-root soybean plants, the total ^13^C accumulation and the ratio of ^13^C accumulation in each organ to the ^13^C accumulation in the whole plant during ^13^CO_2_ feeding (^13^C_ap_) were calculated (**Table 3**). There was no significant difference in ^13^C accumulation in whole soybean plants (Plant_total_), shoots and underground parts (Root_total_) among the three treatments. The ^13^C accumulation in Root_n_ and nodules decreased significantly with increasing nitrate supply concentration on the non-nodulating side, the ^13^C accumulation in the N_200_ treatment was 38.9% (*P*<0.05) and 30.0% (*P*<0.05) lower than that in the N_0_ treatment, respectively. The ^13^C accumulation in Root_non_ increased significantly, and the ^13^C accumulation in the N_100_ and N_200_ treatments was 105.9% (*P*<0.05) and 129.4% (*P*<0.05) higher than that in the N_0_ treatment, respectively.

**Table 3:** ^13^C accumulation and distribution of dual-root soybean plants.

Organs		Shoot	Root				Plant total
			Nodule	Root_n_	Root_non_	Root_total_	
Assimilated C (mg plant^-1^)	N_0_	113.8 ± 3.79 a	6.0 ± 0.44 a	7.2 ± 0.64 a	1.7 ± 0.25 b	14.9 ± 0.52 a	128.7 ± 4.29 a
	N_100_	125.4 ± 8.75 a	5.1 ± 0.15 ab	5.8 ± 0.54 ab	3.5 ± 0.35 a	14.4 ± 1.02 a	139.8 ± 9.59 a
	N_200_	132.0 ± 5.77 a	4.2 ± 0.47 b	4.4 ± 0.09 b	3.9 ± 0.15 a	12.6 ± 0.40 a	144.6 ± 5.43 a
^13^C_ap_ (%)	N_0_	88.4 ± 0.09 b	4.7 ± 0.26 a	5.6 ± 0.50 a	1.3 ± 0.20 b	11.6 ± 0.09 a	100
	N_100_	89.6 ± 0.42 b	3.7 ± 0.23 ab	4.2 ± 0.17 b	2.5 ± 0.15 a	10.4 ± 0.42 a	100
	N_200_	91.2 ± 0.59 a	2.9 ± 0.45 b	3.1 ± 0.15 b	2.7 ± 0.03 a	8.8 ± 0.59 b	100

Root_n_ represented roots on the nodulating side, Root_non_ represented roots on the non-nodulating side, Root_total_ represented the underground parts, Plant_total_ represented whole soybean, ^13^C_ap_ represented the proportion of ^13^C accumulation in each organ to the ^13^C accumulation in the whole plant during ^13^CO_2_ feeding for 8h. Values are means ± standard error (n=3). Different lowercase letters indicate a significant difference between the treatments in the same organ at the 5% level.

With increasing nitrate supply on the non-nodulating side, ^13^C_ap_ values of the shoots increased, and in the N_200_ treatment they were 3.2% higher than that in the N_0_ treatment (*P*<0.05). However, Root_total_^13^C_ap_ decreased with increasing nitrate supply on the non-nodulating side, and the ^13^C_ap_ in the N_200_ treatment was 24.1% lower than that in the N_0_ treatment (*P*<0.05). ^13^C_ap_ values in nodules decreased with increasing nitrate supply concentration on the non-nodulating side, and the value for the N_200_ treatment was significantly lower than that for the N_0_ treatment. ^13^C_ap_ values in Root_n_ samples were consistent with those of nodules, except that the values for both the N_100_ and N_200_ treatments were significantly lower than those for the N_0_ treatment. ^13^C_ap_ values in Root_non_ samples increased with increasing nitrate supply concentration, and the difference between the N_100_ and N_200_ treatments was not significant. However, the values for both treatments were significantly higher than the value for the N_0_ control. These results indicated that the increase in the nitrate supply concentration on the non-nodulating side significantly increased the amount and proportion of ^13^C accumulated in the roots on the non-nodulating side and decreased the amount and proportion of ^13^C accumulated in the roots and nodules on the nodulating side.

Comparing ^13^C_ap_ in each organ of dual-root soybeans, the Shoot/Root_total_ value of the N_200_ treatment was 38.2% (*P*<0.05) and 20.7% (*P*<0.05) higher than the values of the N_0_ and N_100_ treatments, respectively (**Table 4**). The values of (Root_n_+Nodule)/Root_non_, Nodule/Root_non_, and Root_n_/Root_non_ all decreased with increasing nitrate supply on the non-nodulating side. There was no effect on Nodule/Root_n_ values when nitrate was supplied on the non-nodulating side. These results indicated that the assimilated ^13^C distributed to whole soybean roots decreased as the concentration of supplied nitrate on the non-nodulating side increased, the assimilated ^13^C distributed to Root_non_ increased, and the assimilated ^13^C allocated to nodules and Root_n_ decreased. However, the proportion of assimilated ^13^C distributed to nodules and Root_n_ was not affected by the change in nitrate concentration on the non-nodulating side. In summary, fluxes of C to nodules and roots on the nodulating side were suppressed when the concentration of supplied nitrate on the non-nodulating side increased.

**Table 4:** ^13^C accumulation distribution ratio between organs of dual-root soybeans.

Treatments	Shoot/Root_total_	(Root_n_+Nodule)/Root_non_	Nodule/Root_non_	Root_n_/Root_non_	Nodule/Root_n_
N_0_	7.6 ± 0.09 b	8.3 ± 1.21 a	3.7 ± 0.32 a	4.6 ± 0.90 a	0.9 ± 0.12 a
N_100_	8.7 ± 0.35 b	3.1 ± 0.13 b	1.5 ± 0.12 b	1.7 ± 0.03 b	0.9 ± 0.06 a
N_200_	10.5 ± 0.76 a	2.2 ± 0.23 b	1.1 ± 0.15 b	1.2 ± 0.08 b	0.9 ± 0.09 a

Root_n_ represented roots on the nodulating side, Root_non_ represented roots on the non-nodulating side, Root_total_ represented the underground parts. Values are means ± standard error (n=3). Different lowercase letters indicate a significant difference between the treatments at the 5% level.

## 4 Discussion

### 4.1 Relationship between the ultrastructure of soybean nodules and the nitrate supply

Nitrate can be absorbed and transported from one side to the other in dual-root soybeans and inhibits nodule nitrogenase activity systemically (Xia et al., 2017; Lyu et al., 2019; Lyu et al., 2020; Li et al., 2021; Lyu et al., 2022). Infected cells are the main site of N_2_ fixation in nodules. Changes in the characteristics of the infected cells directly affect the N_2_ fixation capacity of nodules (Finan et al., 1983; Domigan et al., 1988). After supplying nitrate, bacteroids in the infected cells of *Chamaecrista fasciculata* nodules became sparse (Naisbitt and Sprent, 1993), newly formed bacteroids lysed and degraded, and viability of mature bacteroids impaired in nodules of barrel medic (Truchet and Dazzo, 1982; Lambert et al., 2020). In this study, when nitrate was supplied to the non-nodulating side for four days, no effect on nodule growth was observed (**Table 1**). However, the ARA and SNA values of the N_100_ and N_200_ treatments decreased by more than 80% compared with those of the N_0_ treatment (**Figure 1**). Meanwhile, with the increase in nitrate supply concentration, the number of infected cells decreased and the number of bacteroids also decreased. The structure of infected cells in the edge part of the nodule close to the roots changed from elongated to spherical and eventually lost regularity in shape at N_200_. Furthermore, numerous vacuoles appeared in the infected cells and became progressively larger (**Figure 2**). When no exogenous N was applied, and N_2_ fixation by nodules was the only source of this macronutrient, no vacuoles in the infected cells of soybean nodules were observed (Gordon et al., 1992). Under environmental stresses (e.g., oxygen limitation or salt stress), large vacuoles were formed around the host cell nucleus of infected cells in senescing soybean nodules. This vacuolization process was accompanied by the lysis of bacteroids (James et al., 1991; James et al., 1993). This is why we indicated that systemic effects of indirect nitrate supply on nodule nitrogenase activity were associated with the changes in nodule structure.

The SM encloses the bacteroids and controls the metabolic fluxes between the plant cytosols and the bacteroids, protecting bacteroids from the attack by host proteases (Streeter, 1991; Day et al., 1995; Udvardi and Day, 1997; Brear et al., 2013; Liu et al., 2018). Meanwhile, iron must cross the SM before being supplied to bacteroids. Iron is used to synthesize ferro proteins necessary for N_2_ fixation, and the oxygen-carrying protein leghemoglobin (Lb) is abundant in nodules (Udvardi and Day, 1997; Brear et al., 2013). In bacteroids, N_2_ fixation is regulated by oxygen (Batut and Boistard, 1994; Fischer, 1994). When the oxygen concentration is not in the correct range, the rhizobia released into the cytoplasm of plant cells can not differentiate into N_2_-fixing bacteria (Brewin, 1989). In this study, the SM was still visibly intact under the N_100_ treatment. However, its less clear outline under the N_200_ treatment may reflect the decomposition of the SM (**Figure 3**). Newcomb et al. (1977) found that reduced N_2_ fixation capacity in ineffective pea nodules was associated with the absence of the SM. The dismantling of the SM is associated with nodule senescence (Fargeix et al., 2004). Thus, changes of the SM structure in this experiment may block iron transport, affect oxygen supply, and cause premature senescence of nodules. A dedicated study with the SM-specific markers is necessary to clarify the fate of the SM in the course of a systemic response to elevated nitrate concentrations.

The PBS became larger with increasing nitrate supply on the non-nodulating side (**Figure 3**). The PBS stores some ions and metabolic substances (Bergersen and Appleby, 1981). Nitrate supply resulted in the enlarged PBS and decreased nodule nitrogenase activity in lupin nodules (De Lorenzo et al., 1990). Salt stress also resulted in the enlarged PBS and decreased nodule nitrogenase activity in fava bean (*Vicia faba* L.) (Trinchant et al., 1998). Although the biological significance of this enlargement of the PBS remains to be elucidated, our findings indicate that the elevated nitrate supply expands the PBS and leads to a decrease in the number of bacteroids. This decrease is accompanied by the fusion of SBs into larger structures, which may explain the expansion of the PBS. Because these ultrastructural rearrangements were associated with the strong suppression of the nitrogenase activity in nodules, we conclude that the microscopic observations caused by treatments N_100_ and N_200_ reflect the progress of N-induced premature nodule senescence.

### 4.2 Relationship between the distribution of assimilated C and the nitrate supply

When nitrate is directly added to roots, the C distribution between roots and nodules in soybean plants changes (Streeter, 1980; Kouchi et al., 1986; Fujikake et al., 2003b; Ishikawa et al., 2018), thus reducing the proportion of C transported to nodules and increasing the proportion of C transported to roots (Fujikake et al., 2003a). Sulieman et al. (2014) also observed similar phenomena in barrel medic. However, this nitrate supply method cannot exclude the toxic effect of nitrate on nodules (Becana et al., 1985; Wasfi and Prioul, 1986; Giannakis et al., 1988; Cabeza et al., 2014). This direct effect of nitrate on nodule growth must result in unequal C partitioning between roots and nodules. In this study, dual-root soybean plants with a single nodulating side were used to supply nitrate to the non-nodulating side, and the nodules on the nodulating side were not exposed to nitrate. With the increase in nitrate concentration, the proportion of assimilated C transported to non-nodulating lateral roots increased significantly, and the proportion of assimilated C transported to nodulating lateral roots and nodules decreased significantly. Moreover, the distribution ratio of assimilated C between nodules and roots on the nodulating side was not influenced by the nitrate concentration supplied on the non-nodulating side (**Table 4**). Previousely, Singleton and Kessel (1987) considered that NH_4_NO_3_ promoted root growth on the N supply side of split-root soybean, reported an increase in the proportion of C distributed to this side as a response to nitrate. We used two varieties of soybeans, nodulating and non-nodulating, for grafting. Due to the difference in varieties, the dry weight of roots on the non-nodulating side was smaller than that on the nodulating side. However, it still showed that more C was transported to the non-nodulating side after nitrate supply. These results indicated that the non-nodulating roots obtained more C than the nodulating side. Roots on the non-nodulating side competed for C with roots and nodules on the nodulating side. Nodulating lateral roots and nodules did not compete for C due to reduced C supply on this side.

PHB is the main C source in the process of bacteroid reproduction and N fixation and can be mobilized to support N fixation when the C supply is insufficient (Wong and Evans, 1971; Bergersen et al., 1991; Trainer and Charles, 2006). The amount of PHB in *C. fasciculata* nodules decreased with increasing nitrate supply concentration (Naisbitt and Sprent, 1993). In this study, the PHB granules became smaller and the hue of PHB granules in TEM images changed from bright to opaque in some bacteroids as the nitrate supply on the non-nodulating side increased (**Figure 3**). It is possible that this observation indicates the onset of PHB degradation to meet the C demand of bacteroids (Bergersen et al., 1991). We interpret this phenomenon as a consequence of the systemic blockage of C flow from the shoot to the nodulated side, which leads to insufficient C supply to nodules. Under the condition of C deficit caused by this systemic response, bacteroids probably intensify the utilization of deposited PHB. However, this mobilization of stored C does not compensate for the reduction in C influx *via* the nodule vasculature, which is manifested in the strongly reduced nitrogenase activity.

## 5 Conclusions

In a dual-root soybean system, where only one side has the ability to develop nodules, nitrate supply to the non-nodulating side systemically suppressed nodule nitrogenase activity, which was related to the change in nodule ultrastructure. Moreover, the two root systems competed for assimilated C from the shoots, and there is no C competition between roots and nodules without direct exposure to nitrate. Although the non-nodulating roots received consistently less photosynthetic C in the control N_0_ and in all the treatments (N_100_ and N_200_) compared with the nodulating side, with the increase in the concentration of applied nitrate, the symbiotic side received progressively less C with the following trend: N_0_ > N_100_ >N_200_. However, for the non-symbiotic side, the trend was the opposite: N_0_ < N_100_< N_200_.

## Data availability statement

The original contributions presented in the study are included in the article/**Supplementary Material**. Further inquiries can be directed to the corresponding author.

## Author contributions

All authors participated in the conception and the design of the study. SL and ZG designed the research. XL and CY conducted the research. FX, HoL and XW contributed in the experiment sampling. CM and SZ provided financial support. ZL revised the language. SL analyzed the data and wrote the manuscript. CW and HL revised the manuscript. All authors contributed to the article and approved the submitted version.
