# Supplementary material for: Systemic regulation of nodule structure and assimilated carbon distribution by nitrate in soybean
Source: Front Plant Sci. 2023 Feb 6;14:1101074. doi: 10.3389/fpls.2023.1101074 (PMC9939697; doi:10.3389/fpls.2023.1101074)
Supplement: Supplementary file 1 [file DataSheet_1.doc]

**S1 Preparation of Dual Root Soybean Systems with single side nodulating**

The experiments were conducted in sand culture. Prepared plastic pots of 30 cm in diameter and 28 cm in height, which were divided into two equal parts in the middle with a custom-made polycarbonate plastic partition plate. The gap between the plate and the pot was sealed with glue. Two drainage holes 1 cm in diameter were drilled at the bottom of the pot. Each pot was filled with 20 kg of sand.

Seeds of nodulating soybeans (*Glycine max* L. cv. Dongda ) and non-nodulating soybeans (*Glycine max* L. cv. WDD01795, L8-4858) were seeded into fine-sand medium and cultured in an illuminated growth chamber at 30°C for approximately 3 days. When the distance between the growing point of the cotyledon and the tip of the root was 7 to 10 cm, the roots of the soybean seedlings were rinsed with water and then used for grafting. Using a sterilization blade to make a 0.5-1.0cm long incision (not cut off) up (nodulating seedlings) or down (non-nodulating seedlings) at the upper middle of the hypocotyls of the seedlings. the non-nodulating roots noted as root_non_, and nodulating roots noted as root_n_ (Fig. S1A). the two seedlings were cross-inserted into their cuts (Fig. S1B) and clipped with a grafting clamp (Fig. S1C). The roots of the two seedlings were planted into fine-sand medium of each half of the pot divided by the partition, and the grafting site was just above the partition plate. Then the dual-root seedlings were fastened with transparent plastic cups with vents to prevent the effects of rain on grafted seedlings (Fig. S1D). A week later, removed the plastic cup and grafting clamps, then cut off the shoot of non-nodulating seedling from the grafting, so that the seedling became a whole with two roots sharing the nodulating shoot (Fig. S1E, F).


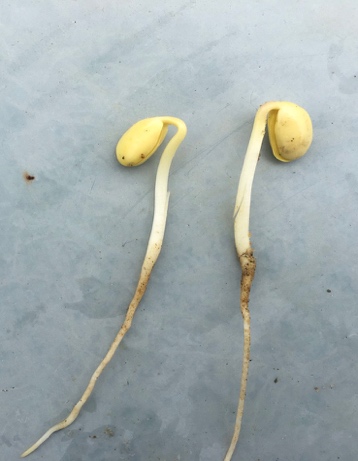

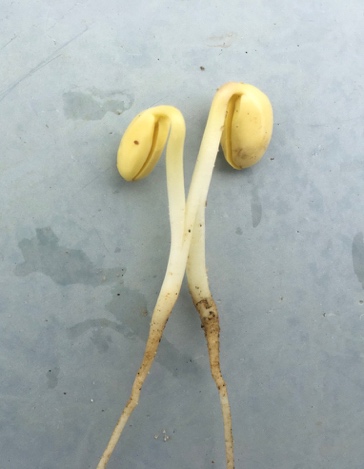

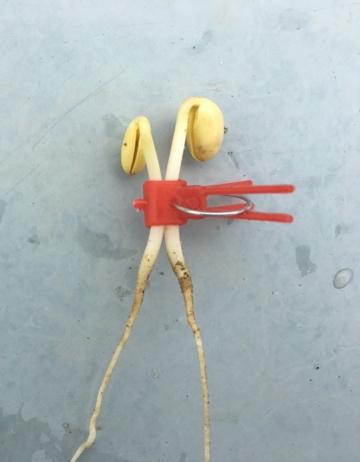


B

A

C

**Root_non_**

**Root_n_**

*
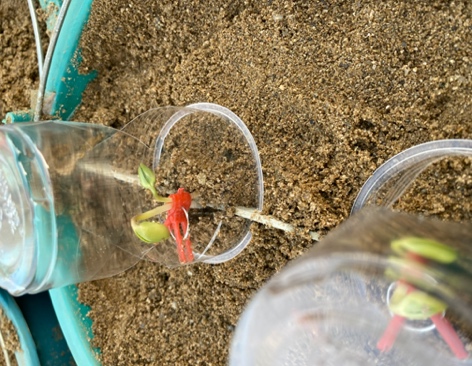
*
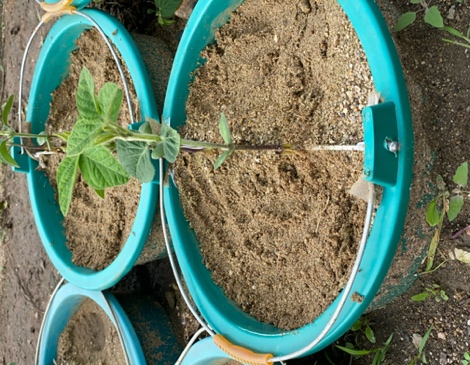
 *
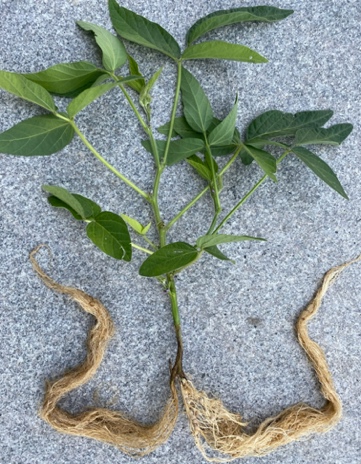
*

D

E

F

**Root_non_**

**Root_n_**

**S2 Concentration** of N-free nutrient solution of sand culture

| Inorganic salts | Concentration（mg/L） | Inorganic salts | Concentration（mg/L） |
| --- | --- | --- | --- |
| KH_2_PO_4_ | 136.00 | ZnSO_4_·7H_2_O | 0.22 |
| MgSO_4_ | 240.00 | MnCl_2_·4H_2_O | 4.90 |
| CaCl_2_ | 220.00 | H_3_BO_3_ | 2.86 |
| Na_2_MoO_4_·H_2_O | 0.03 | Fe–EDTA* |  |
| CuSO_4_·5H_2_O | 0.08 |  |  |

*Note: The solution which contain 5.57 g FeSO_4_·7H_2_O and 7.45 g Na_2_EDTA per one L respectively were added into nutrient medium as the rate of 1:1000 when using.

**S3 The ^13^CO_2_ assimilation device**

**
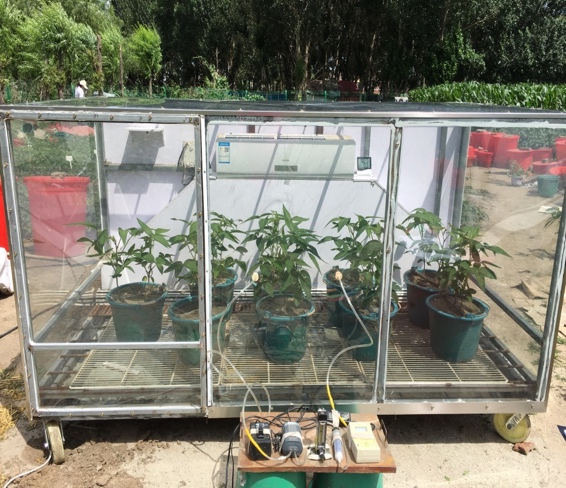

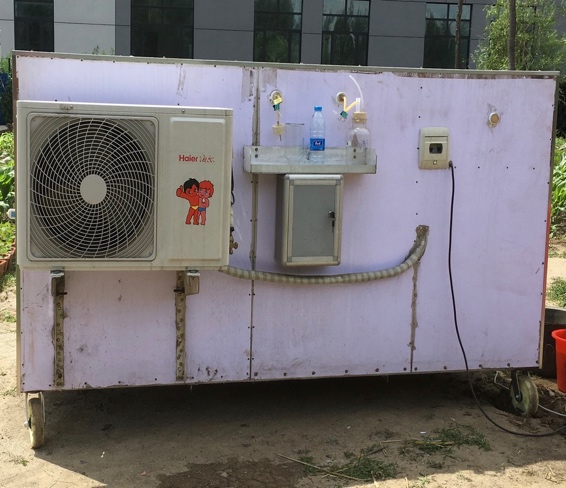
**

infrared carbon

dioxide analyzer

CO_2_ generated device

**S4 Total carbon concentration of dual-root soybean plants (g kg^-1^ plant^-1^**)

| Treatments | Shoot | Root | | | | Plant total |
| --- | --- | --- | --- | --- | --- | --- |
|  |  | Nodule | Root_n_ | Root_non_ | Root_total_ |  |
| N_0_ | 420.2±3.39 a | 445.3±1.98 a | 397.7±2.82 a | 366.2±7.20 b | 1209.7±8.01 ab | 1630.±8.08 ab |
| N_100_ | 423.5±1.12 a | 463.8±9.38 a | 383.9±12.38 a | 395.4±9.92 a | 1243.7±16.29 a | 1667.3±16.86 a |
| N_200_ | 421.5±1.68 a | 465.9±8.73 a | 355.9±4.81 b | 368.1±2.51 b | 1189.3±7.31 b | 1610.7±8.67 b |

Note: Root_n_ represented roots on nodulating side, Root_non_ represented roots on non-nodulating side, Root_total_ represented the underground parts, Plant_total_ represented whole soybean. Values are means ± standard error (n=3). Different lowercase letters indicate a significant difference between the treatments at the 5% level.
